# Effects of Exendin-4 on bone marrow mesenchymal stem cell proliferation, migration and apoptosis *in vitro*

**DOI:** 10.1038/srep12898

**Published:** 2015-08-07

**Authors:** Hao Zhou, Dandan Li, Chen Shi, Ting Xin, Junjie Yang, Ying Zhou, Shunyin Hu, Feng Tian, Jing Wang, Yundai Chen

**Affiliations:** 1 Department of Cardiology, Chinese PLA General Hospital, Beijing, China; 2 Department of Radiotherapy, Beijing Cancer Hospital, Beijing, China; 3 Department of Cardiology, Tianjin First Central Hospital, Tianjin, China

## Abstract

Mesenchymal stem cells (MSC) are regarded as an attractive source of therapeutic stem cells for myocardial infarction. However, their limited self-renewal capacity, low migration capacity and poor viability after transplantation hamper the clinical use of MSC; thus, a strategy to enhance the biological functions of MSC is required. Exendin-4 (Ex-4), a glucagon-like peptide-1 receptor agonist, exerts cell-protective effects on many types of cells. However, little information is available regarding the influence of Ex-4 on MSC. In our study, MSC were isolated from bone marrow and cultured *in vitro.* After treatment with Ex-4, MSC displayed a higher proliferative capacity, increased C-X-C motif receptor 4 (CXCR4) expression and an enhanced migration response. Moreover, in H_2_O_2_-induced apoptosis, Ex-4 preserved mitochondrial function through scavenging ROS and balancing the expression of anti- and pro-apoptotic proteins, leading to the inhibition of the mitochondria-dependent cell death pathways and increased cell survival. Moreover, higher phospho-Akt (p-Akt) expression was observed after Ex-4 intervention. However, blockade of the PI3K/Akt pathway with inhibitors suppressed the above cytoprotective effects of Ex-4, suggesting that the PI3K/Akt pathway is partly responsible for Ex-4-mediated MSC growth, mobilization and survival. These findings provide an attractive method of maximizing the effectiveness of MSC-based therapies in clinical applications.

Myocardial infarction induces the irreversible loss of cardiomyocytes and scar formation, which ultimately results in congestive heart failure. Bone marrow mesenchymal stem cells (MSC) are multipotent adult stem cells that can regenerate injured heart tissue through differentiation into many types of cells and production of paracrine cytokines^1^. Both animal and clinical studies have shown^2^,^3^,^4^ that MSC transplantation can improve left ventricular ejection fraction, reduce infarct size and reverse cardiac remodeling. However, many challenges limit the use of MSC-based therapy. First, adult stem cells undergo fewer replicative cycles compared with embryonic stem cells *in vitro*^5^, which may hinder the harvesting of a sufficient number of stem cells for adequate therapy^6^. Next, transplanted cells undergo apoptosis triggered by oxidative stress^7^ or washout from the heart caused by the loss of homing factors, such as chemokine C-X-C motif receptor 4 (CXCR4)^8^, leading to low rates of MSC survival and retention in the infarcted area^9^. Thus, methods to advance the proliferation, migration and anti-apoptotic capacity of MSC are required to improve the efficiency of MSC transplantation^10^,^11^,^12^. The PI3K/Akt signaling pathway has been reported to play an important role in MSC growth^13^, mobilization^14^ and survival^15^. Therefore, several strategies have been proposed to active the PI3K/Akt pathway, including genetic modification or preconditioning with drugs. Although these attempts have obtained exciting and promising results, the high cost and potential side effects of these strategies have limited their applications.

Exendin-4 (Ex-4), a novel antidiabetic agent isolated from the salivary glands of the Gila monster, shares 53% homology with human glucagon-like peptide-1 (GLP-1) and is primarily designed to improve glucose control by acting on the glucagon-like peptide-1 (GLP-1) receptor (GLP-1R)^16^. However, researchers have recently found that Ex-4 also promotes the proliferation or survival of many types of cells^17^,^18^ via GLP-1R-dependent and -independent pathways^19^,^20^. However, little information is available regarding the effects of Ex-4 on MSC biological functions. In this study, we observed beneficial effects of Ex-4 on MSC proliferation, migration and survival. We then examined whether the modulatory role of Ex-4 in MSC was due to the activation of the PI3K/Akt signaling pathway.

## Materials and Methods

### Ethics statement

The present study was conducted in accordance with the Declaration of Helsinki and the guidelines of the Ethics Committee of Chinese PLA (People’s Liberty Army) General Hospital, Beijing, China. The all experimental protocol were approved by Ethics Committee of Chinese PLA (People’s Liberty Army) General Hospital, Beijing, China.

### Isolation, culture and identification of bone marrow MSC

Bone marrow MSC were isolated and harvested as previously described. In brief, bone marrow samples from Sprague-Dawley rats were diluted with PBS and centrifuged at 400 × g for 30 min. The buffy coat containing mononuclear cells was washed twice with PBS and plated in MSC culture growth medium, which consisted of Dulbecco’s modified Eagle’s medium (DMEM; Invitrogen Co., USA) with L-glutamine and 10% (v/v) fetal bovine serum (FBS). Cultures were maintained at 37 °C in a humidified atmosphere containing 5% carbon dioxide. After 24 h, non-adherent cells were discarded, and fresh medium was added and replaced every 4 days. Each primary culture was subcultured at a 1:2 ratio when the MSC reached approximately 80% confluence.

For adipogenic and osteogenic differentiation, StemPro® adipogenesis and osteogenesis differentiation media (Invitrogen Co., USA) were used according to the manufacturer’s protocol. Cultures were refreshed every 2 to 3 days. After 3–4 weeks of culture, adipogenesis was assessed by incubating cells with Oil Red O solution to stain neutral lipids in the cytoplasm. Alizarin Red S (Sigma-Aldrich, USA) was used to evaluate osteogenic differentiation.

To confirm MSC characterization, cultured cells at passage 3 were subjected to flow cytometry using FITC-labeled CD29, CD31, CD34, CD45, CD73, CD90, CD105 and CD166 markers (BD Biosciences, USA) at the manufacturer-recommended concentrations. Cells stained with FITC-labeled IgG were used as negative controls. The analysis used a FACScan for at least 10,000 events using CellQuest software.

### Cell viability assay

MSC cell viability was assessed using the 3-(4,5-dimethylthiazohl-2-yl)-2,5-diphenyltetrazolium bromide (MTT; Sigma-Aldrich, USA) assay. Briefly, MSC were seeded in 96-well plates. After treatment with different doses of Ex-4 (0–20 nm/L, Sigma-Aldrich) for 12 h, the cells were incubated with MTT solution (Sigma-Aldrich) at 37 °C for 4 h. Then, the medium was removed, and 100 μl of dimethyl sulfoxide (DMSO) was added to each well. The absorbance was measured at a wavelength of 570 nm. The data are expressed as the ratio of the optical density (OD) value of the treated group to the OD of the control group.

### Flow cytometry

The CXCR4 expression, apoptotic rate and cell cycle distribution of MSC were evaluated using flow cytometry. After treatment with different concentrations of Ex-4 (0–20 nm/L) for 24 h, MSC at P_3_ were used to detect CXCR4 expression. After being washed in PBS, each group of cells was stained with FITC-labeled CXCR4 (Bioss, China) or FITC-labeled IgG for 30 min at room temperature.

The number of apoptotic cells was analyzed quantitatively using the Annexin V–FITC/PI Apoptosis Detection Kit (BD Biosciences, USA). After treatment, the cells were harvested, resuspended in 200 μl of binding buffer, and then incubated with 5 μl of Annexin V–FITC/binding buffer mixture (30 min, 37 °C) in the dark. Subsequently, the cells were incubated with 10 μl of propidium iodide for 5 min and immediately analyzed by bivariate flow cytometry using a BD FACSCalibur cytometer.

For the cell cycle analysis, MSC were treated with Ex-4 (0–20 nm/L) for 24 h. The cells were then resuspended in PBS and fixed with ice-cold 70% ethanol for 24 h. The fixed cells were rinsed and resuspended in 50 μg/ml propidium iodide for 30 min in the dark. Flow cytometric analyses were performed using a BD FACSCalibur cytometer.

### Cell proliferation assay

Cell proliferation was assessed with the cell counting kit-8 (CCK-8) assay (Beyotime Institute of Biotechnology, China) and 5-ethynyl-2´-deoxyuridine (EdU) proliferation assay (RiboBio Co., China). For the CCK-8 test, cells were plated onto 96-well plates (5 × 10^3^ cells/well) with Ex-4 (0–20 nm/L) in a triplicate pattern. Assays were performed from 1 to 7 days after plating by the addition of 100 μl of fresh medium in 10 μl of the CCK-8 solution for another 2 h at 37 °C. The OD at 570 nm was measured. The assay was repeated 3 times.

The EdU assay (RIBOBio Co, Guangzhou, China) was used to measure cells’ ability to proliferate after treatment with 20 nm Ex-4 for 24 h. After incubation with EdU for 2 h, the cells were fixed with 4% paraformaldehyde and permeabilized with 0.5% Triton X-100. Then, the Apollo^®^ reaction cocktail (reaction buffer and Apollo^®^ 643 fluorescence) was added to the medium for another 30 min in the dark. After being washed with PBS 3 times, the cells were stained with DAPI (Sigma-Aldrich) for 5 min and immediately viewed under fluorescence microscopy. The number of EdU^+^ cells was calculated by counting at least three random separate fields.

### Western bolt analysis

Following the appropriate treatments, cultured cells were lysed with RIPA lysis buffer (Beyotime, China) for 30 min, followed by centrifugation at 14,000 × g for 30 min. The protein concentration of each sample was quantified with the BCA protein assay (Beyotime Institute of Biotechnology, China). Then, equal amounts of proteins were electrophoresed on a 6% to 15% gradient gel by sodium dodecyl sulfate–polyacrylamide gel electrophoresis and transferred to a polyvinylidene difluoride membrane. The membranes were blocked with 5% milk in Tris-buffered saline and 0.2% Tween at room temperature for 1 h and then incubated overnight at 4 °C with the following specific primary antibodies: β-actin (1:2000), caspase3 (1:2000), pro-caspase3 (1:1000), caspase9 (1:1000), c-IAP1 (1:1000), t-Akt (1:2000) and p-Akt (1:1500), purchased from Cell Signaling Technology, USA; p-Rb (1:2000), Bcl-2 (1:2000), Bax (1:2000), c-IAP2 (1:1000) survivin (1:1500) and Bad (1:1000), purchased from Abcam, USA; and Cyclin D1 (1:1500), Cyclin E (1:1500) and CXCR4 (1:500), purchased from Santa Cruz Biotechnology, USA. The blots were washed three times with Tris-buffered saline and 0.2% Tween and incubated with a horseradish peroxidase-conjugated secondary antibody (Santa Cruz Biotechnology) for 45–60 min at room temperature. The expression signals were detected with an enhanced chemiluminescence (ECL) reagent (Beyotime Institute of Biotechnology, China) after the membranes were washed with TBST (10 min× 3).

### Migration and wound-healing assays

The MSC migration assay was performed using a 24-well transwell chamber with a pore size of 8 μm (Corning, USA). First, MSC were cultured with Ex-4 (0-20 nm/L) for 24 h; then, 10^5^ MSC were seeded into the upper chamber in serum-free medium. Stromal cell-derived factor-1α (SDF-1, Sigma-Aldrich, 50 ng/ml) was added to the lower chamber. After a 12-h incubation at 37 °C, the non-migrating cells in the upper chamber were carefully removed with a cotton swab, and the cells that had traversed the membrane were fixed in methanol and stained with 0.05% crystal violet. For quantification, the number of migrated cells was calculated by counting at least five random separate fields as the ratio of the experimental samples to the control samples × 100.

For the wound-healing assay, 10^6^ cells were seeded onto 36-mm plates and grown to 80–90% confluency. An artificial wound was created using a P200 pipette tip to scratch the confluent cell monolayer. Photomicrographs were immediately obtained (time 0 h), and then, the cells were incubated in DMEM containing 1% fetal bovine serum with or without Ex-4 for 24 h. The mobilization of the cells and closing of the scratch wound was observed 24 h later.

### Oxidative stress injury induction and TUNEL assay

Oxidative stress apoptosis in MSC was induced by hydrogen peroxide and serum deprivation. Briefly, the MSC medium was replaced with serum-free DMEM supplemented with 0.3 mM H_2_O_2_, and the MSC were incubated at 37 °C under normoxic conditions for 12 h. For the Ex-4 protection experiments, MSC were pretreated with Ex-4 (0–20 nM) for 12 h; then, the medium was discarded and replaced with serum-free DMEM supplemented with 0.3 mM H_2_O_2_ for another 12 h. The terminal deoxynucleotidyl transferase-mediated dUTP-biotin nick end labeling (TUNEL) assay was used to detect the apoptosis of MSC under H_2_O_2_ according to the manufacturer’s protocol. The degree of apoptosis was calculated as the number of TUNEL-positive cells per 500 MSC nuclei. The nuclei were stained with the chromatin dye DAPI. Briefly, cells were fixed for 1 h in 4% (w/v) paraformaldehyde at room temperature. After specific labeling was performed, the cells were exposed to DAPI in the dark for 5 min. Then, the immunostained MSC were observed by fluorescence microscopy.

### Caspase3 activity, ROS, MDA, SOD, GSH, and GPX assays

Because the activation of caspase3 represents an essential step in the apoptotic process, an Ac-DEVD-AMC caspase3 fluorogenic substrate (Beyotime Institute of Biotechnology, China) caspase3 activity kit was used to detect caspase3 activity according to the manufacturer’s protocol. The relative caspase3 activity was calculated as the ratio of emission of treated cells to untreated cells. The assay was repeated 3 times.

Dihydroethidium (DHE; Invitrogen, Germany) was used to detect intracellular ROS, and 10 μM DHE was added to the cell culture medium, which was then incubated in the dark and viewed under laser confocal microscopy (Olympus).

Malondialdehyde (MDA) is an end product of lipid peroxidation that occurs as a result of oxidative damage and is a reliable marker of the level of damage to MSC caused by H_2_O_2_. Glutathione (GSH), glutathione peroxidase (GPX) and superoxide dismutase (SOD) are important antioxidants involved in the effective scavenging of free radicals and in suppressing the actions of oxidative stress, which promotes the survival of MSC exposed to H_2_O_2_. MDA content, SOD/GPX activity and GSH concentration were measured using commercial kits (Beyotime Institute of Biotechnology, China) following the manufacturer’s instructions.

### Reagent treatment

To investigate the role of the PI3K/Akt pathway in Ex-4-mediated MSC proliferation, migration and survival, LY294002 (20 μm/L, Cell Signaling Technology) was added to the MSC medium for 4 h before Ex-4 treatment to block PI3K/Akt pathway activation. For the proliferation experiments (cell cycle analysis and EdU assay), MSC were treated with Ex-4 for 24 h before the above assays. For the chemotaxis assay, MSC were cultured with Ex-4 for 24 h and then seeded in chemotaxis chambers for 12 h. Moreover, to block the interaction between CXCR4 and SDF-1α, MSC were incubated with or without CXCR4 antibody (AMD3100, Abcam, USA, 10 μg/ml) for 2 h before Ex-4 treatment (20 nm/L). In the H_2_O_2_-induced apoptosis model, MSC were pretreated with Ex-4 (0–20 nm/L) for 12 h and then incubated with 0.3 mM H_2_O_2_ for 12 h.

### Statistical analyses

The results are shown as means the ± SDs of at least 3 replicates. For group-wise comparisons, a one-way ANOVA with Scheffe’s post-hoc correction was performed using SPSS 17. Values at *P* < 0.05 were considered significant.

## Results:

### MSC morphology and characterization

**Figure 1:**
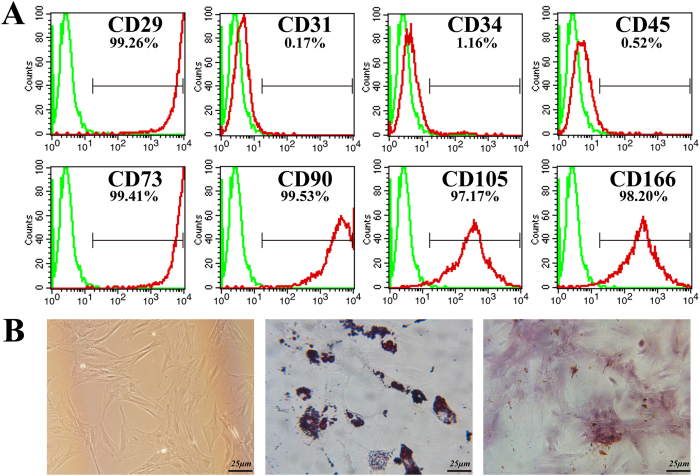
MSC characterization. (**A**) Flow cytometry results demonstrated that MSC were uniformly negative for CD31, CD34 and CD45 and positive for CD29, CD73, CD90 CD105 and CD166 expression. (**B**) Isolated MSC showed fibroblast-like shapes and exhibited multi-differentiation capacity. Bar, 25 μm.

The MSC cultured in medium demonstrated a spindle-like or fibroblast-like shape with directionality and regularity. MSC were characterized by surface marker expression via FACS analysis. The results presented in Fig. 1A indicate that the MSC expressed CD29, CD73, CD90, CD105, and CD166, but these cells did not express the endothelial marker CD31 or the hematopoietic lineage markers CD34 and CD45, which was in agreement with previous reports. A differentiation assay was used to evaluate the multi-differentiation capacity of MSC. After several days of induction toward the adipogenic lineage, considerable morphological changes with lipid vacuole accumulation were observed (Fig. 1B). Following 21 days of osteogenic differentiation, positive staining for Alizarin Red S confirmed the differentiation of MSC into osteocytes.

### Effects of Ex-4 on MSC cellular viability and the PI3K/Akt pathway

**Figure 2:**
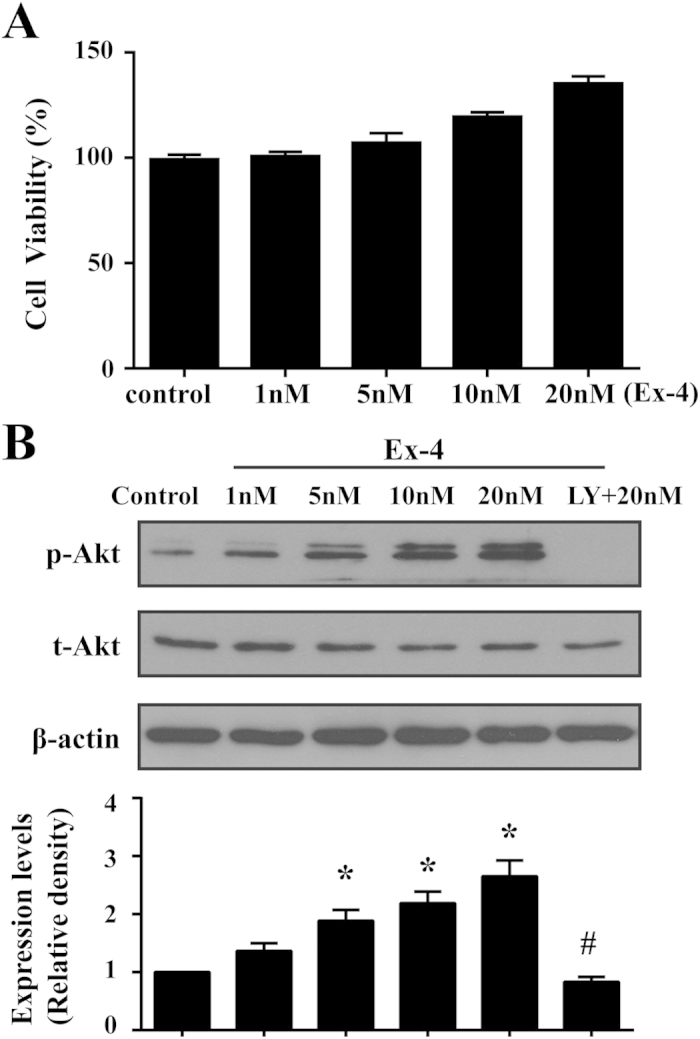
The effects of Ex-4 on viability and the PI3K/Akt pathway in MSC. MSC were incubated with different doses of Ex-4 for 24 h. Their viability was assessed with MTT, and the activation of the PI3K/Akt pathway was evaluated by western blotting. (**A**) Ex-4 had no toxic effects on MSC. (**B**) Ex-4 activated Akt in a dose-dependent manner. *P < 0.05 vs. control group, ^#^*P* < 0.05 vs. Ex-4 group; LY, LY294002.

First, we assessed whether Ex-4 itself had toxic effects on MSC. As shown in Fig. 2A, over the entire range of concentrations used (1–20 nM), Ex-4 had little influence on cell viability compared with untreated cells, indicating that Ex-4 had no toxic effects on MSC.

It has been confirmed that Ex-4 exerts cytoprotective effects on islet MECs through the ERK1/2, cAMP/PKA, and PI3K/Akt pathways^21^, which mediate classical growth and survival signals in MSC 22. Thus, we examined the effects of Ex-4 on the PI3K/Akt pathway in MSC. As shown in Fig. 2B, Ex-4 treatment increased the levels of phosphorylated Akt (p-Akt) in a concentration-dependent manner. In the presence of PI3K/Akt inhibitors, however, p-Akt expression was strongly inhibited, indicating that Ex-4 was the upstream activator of the PI3K/Akt pathway in MSC.

### PI3K/Akt was required for Ex-4-induced proliferation

**Figure 3:**
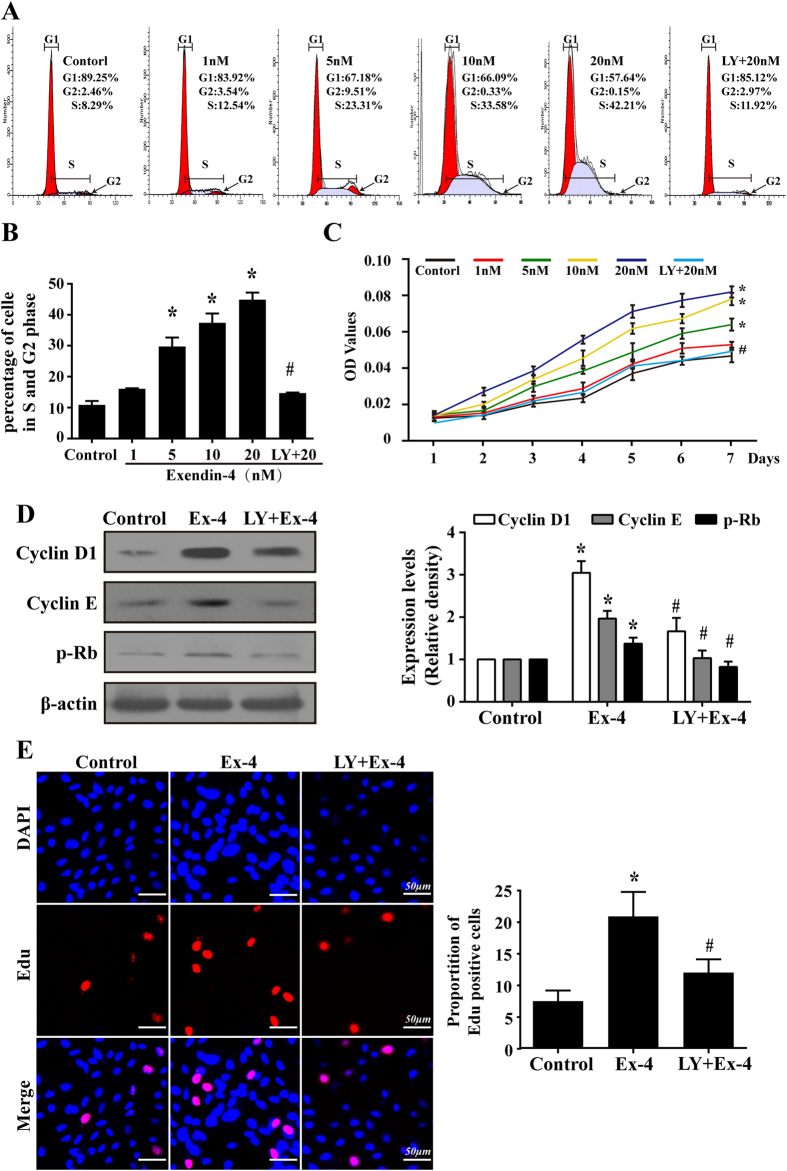
The PI3K/Akt signaling pathway was required for the MSC proliferation induced by Ex-4. (**A** and **B**) MSC were treated with Ex-4 (0–20 nM) for 24 h, and the cell cycle was then evaluated using flow cytometry. (**C**) Growth curve of MSC after Ex-4 (0–20 nM) intervention from days 1 to 7. (**D**)After treatment with Ex-4 for 24 h, the protein expression levels of Cyclin D1, Cyclin E, and p-Rb were increased; however, they were decreased by the pre-inhibition of the PI3K/Akt pathway. (**E**) The EdU assay showed that a blockade of PI3K/Akt could reduce Ex-4-mediated MSC proliferation. *P < 0.05 vs. control group; ^**#**^*P* < 0.05 vs. Ex-4 group; LY, LY294002.

To establish the role of Ex-4 in MSC growth, varying doses of Ex-4 were added to the culture medium for 24 h, and then, the cell cycle distribution was analyzed by flow cytometry. As shown in Fig. 3A,B, an increased fraction of cells of the Ex-4 group were in the S and G_2_/M phases, and fewer cells were in the G_1_ phase of the cycle, suggesting that Ex-4 caused a dose-dependent increase in the number of cells undergoing division. To further confirm whether the promotion of the cell cycle by Ex-4 would increase the cellular life span, the effects of Ex-4 on the growth kinetics of MSC at a specified point were assessed using CCK-8. The growth curves shown in Fig. 3C revealed that the growth ability of the MSC improved gradually with increases in the Ex-4 concentration. However, PI3K/Akt inhibition suppressed these effects of Ex-4 on MSC proliferation. Moreover, there were no substantial differences between the control group and the 1-nM group, and, during the first 24 h, no significant differences were found between the groups.

Next, we found that the expression levels of Cyclin D1, Cyclin E and p-Rb (Fig. 3D) increased with Ex-4 treatment but were reduced by blockade of PI3K/Akt. Cyclin D1 and Cyclin E form the Cdk4/6-cyclin D and Cdk2-cyclin E complex^23^, which plays a key role in the cycle transition from the G_0_/G_1_ to S phases via the phosphorylation of Rb (p-Rb). The release of p-Rb activates the transcription factor E2F-1, which allows the expression of genes that are necessary for DNA replication and mitosis^24^. Furthermore, the EdU proliferation assay (Fig. 3E) displayed similar results: more EdU-positive cells were in the Ex-4 group (20 nM for 24 h) than in the control group. However, pretreatment with LY294002 reversed this trend, suggesting that Ex-4 increased the proliferative capacity of MSC through the PI3K/Akt pathways, which promoted the cell cycle via a Cyclin D1/E-Rb-dependent pathway.

### Ex-4 enhanced CXCR4 expression and the subsequent MSC migration via PI3K/Akt

**Figure 4:**
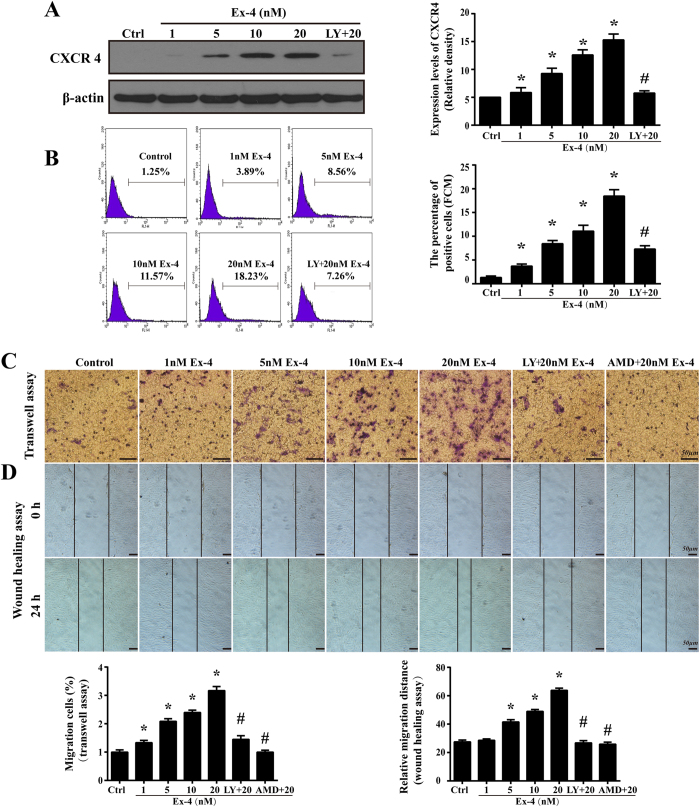
Ex-4 improved CXCR4 expression and migration in MSC via PI3K/Akt. (**A**) CXCR4 expression after Ex-4 (0–20 nM) treatment for 24 h was analyzed by western blotting. (**B**) The flow cytometry was used to quantificational measure the CXCR4 expression. The values on the plots are representative of one experiment. (**C**) The MSC migration response to SDF-1 was analyzed using a transwell assay. (**D**) A wound-healing assay was conducted to detect cell motility after MSC were stimulated with or without Ex-4. Bar, 50 μm; *P < 0.05 vs. control group; ^**#**^*P* < 0.05 vs. Ex-4 group; LY, LY294002; AMD, AMD3100.

SDF-1α and its unique receptor CXCR4 play a key role in the mobilization and recruitment of MSC to injured heart tissue after transplantation^25^. In our study, we found little expression of CXCR4 in normal MSC, whereas Ex-4 increased CXCR4 protein levels, which reached a maximum at 20 nM Ex-4 (Fig. 4A). Furthermore, the upregulation of intracellular CXCR4 expression levels resulted in an increase in cell surface expression, which was evidenced by more CXCR4-positive cells in the Ex-4 group than in the control group (18.46 ± 1.33% in the 20 nM group vs. 1.31 ± 0.32% in the normal group, *P* < 0.05, Fig. 4B). However, pretreatment with a PI3K/Akt inhibitor partly reduced both the intracellular expression and the cell surface expression of CXCR4 (Fig. 4A,B), suggesting that the upregulation of CXCR4 in the Ex-4-treated MSC was attributable to the PI3K/Akt pathways.

To determine whether altered CXCR4 expression in MSC results in a heightened migration capacity, we performed a transwell assay to investigate the chemoattractive response of Ex-4-treated MSC. As shown in Fig. 4C, exposing MSC to Ex-4 for 24 h caused more cells to translocate through the insert membrane in a concentration-dependent manner, indicating that MSC with higher CXCR4 expression held an increased migratory response. Correspondingly, the inhibition of PI3K/Akt resulted in fewer cells located in the lower chamber, suggesting that the heightened chemoattractive response under Ex-4 was PI3K/Akt dependent. The wound-healing assay also supported the above findings that Ex-4 promoted MSC motility via PI3K/Akt (Fig. 4D). We also found that blocking the SDF-1/CXCR4 interaction with AMD3100 completely abolished the migration response of MSC, showing that SDF-1/CXCR4 was necessary for MSC migration.

### Role of Ex-4 and Akt in H_2_O_2_-induced MSC apoptosis

**Figure 5:**
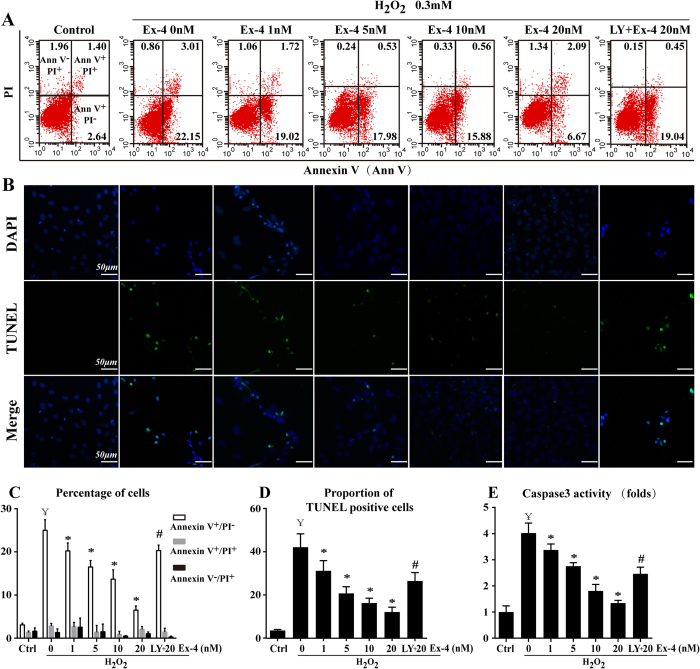
PI3K/Akt was involved in the anti-apoptotic effects of Ex-4 on MSC under oxidative stress. MSC were pretreated with Ex-4 (0–20 nM) for 24 h and were then incubated with 0.3 mM H_2_O_2_ for 12 h. Cell apoptosis were confirmed by Annexin V/PI (**A**,**C**) and TUNEL staining (**B**,**D**). (**E**) Caspase3 activity assay. ^¥^*P* < 0.05 vs. control group; *P < 0.05 vs. H_2_O_2_ group; ^#^*P* < 0.05 vs. Ex-4 group; LY, LY294002.

Our previous research demonstrated that Ex-4 protected adipose-derived mesenchymal stem cells from oxidative injury and that PI3K/Akt was involved in the anti-apoptotic effect of Ex-4^26^. However, the effects and mechanisms of Ex-4 on MSC apoptosis remain unclear. Under our experimental conditions, H_2_O_2_ was applied to induce MSC apoptosis. We found that approximately 25.01 ± 2.48% of the cells in the H_2_O_2_ group were Annexin V^+^/PI^−^. However, a significant concentration-dependent reversal of the H_2_O_2_-induced Annexin V^+^/PI^−^ cells was observed when stressed cells were treated with Ex-4 (Ex-4 1 nM, 20.25 ± 1.84%; 5 nM, 16.51 ± 1.51%; 10 nM, 13.68 ± 2.16%; 20 nM, 6.51 ± 0.95%; *P* < 0.05 vs. H_2_O_2_; Fig. 5A,C), suggesting that Ex-4 abolished the effects of H_2_O_2_ on early apoptosis (Annexin V^+^/PI^−^). However, Ex-4 had no effects on late apoptosis (Annexin V^+^/PI^+^; *P *> 0.05 vs. H_2_O_2_) or necrosis (Annexin V^−^/PI^+^; *P *> 0.05 vs. H_2_O_2_). Furthermore, DNA damage caused by oxidative stress inevitably induces cells to progress to apoptosis or death. Thus, the TUNEL assay was used to confirm the presence of DNA fragmentation (Fig. 5B,D). The analysis showed a concentration-dependent decrease in TUNEL^+^ cells when MSC were exposed to Ex-4 prior to H_2_O_2_. In addition, because the activation of caspase3 represents an essential step in the apoptotic process, we evaluated caspase3 activity. The results showed that the activity of caspase3 increased in the H_2_O_2_ group but decreased when MSC were pretreated with Ex-4 (Fig. 5E). Preconditioning with LY294002 to block Akt caused the protective effects of Ex-4 on H_2_O_2_-treated MSC to disappear, as evidenced by an increase in the number of apoptotic cells and higher caspase3 activity. These results indicated that Akt was partly responsible for the anti-apoptotic role of Ex-4 in H_2_O_2_-induced MSC death.

### The anti-apoptotic effect of Ex-4 was attributed to ROS scavenging and the preservation of mitochondrial function via the PI3K/Akt pathway

**Figure 6:**
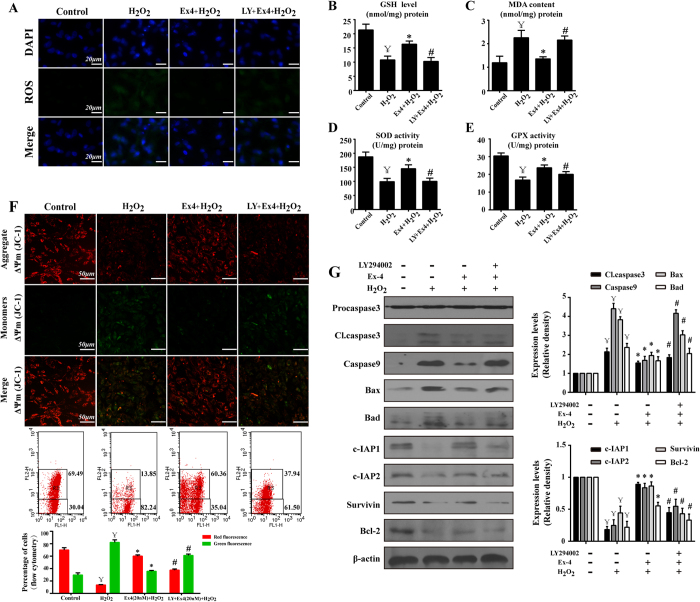
Effects of Ex-4 on ROS and the mitochondrial apoptosis pathway. MSC were treated with 20 nM Ex-4 for 24 h and then with H_2_O_2_ for 12 h. (**A**) Cellular ROS was assessed with ROS-DHE. (**B–E**) Changes in MDA, GSH, SOD and GPX levels after Ex-4 treatment with or without LY294002. (**F**) The mitochondrial transmembrane potential of MSC was analyzed by flow cytometry and immunofluorescence. In the flow cytometry analysis, R3 (upper regions) represented distribution red fluorescence, and the lower regions represented green fluorescence. Normal ΔΨm exhibited red fluorescence, indicating coupled mitochondria, whereas reduced ΔΨm displayed green fluorescence. (**G**) Changes in the expression of proteins associated with mitochondrial apoptosis pathways. Blue indicates cell nuclei; ^¥^*P* < 0.05 vs. control group; *P < 0.05 vs. H_2_O_2_ group; ^#^*P* < 0.05 vs. Ex-4 group; LY, LY294002.

Oxidative stress can result from increased ROS content, and excessive ROS attack mitochondria, leading to reduced mitochondrial membrane potential (ΔΨm), which initiates cellular apoptosis^27^. In our experiment, exogenous H_2_O_2_ observably produced more intracellular ROS, but Ex-4 preconditioning rapidly and almost completely scavenged most of it (Fig. 6A). Additionally, Akt inhibition suppressed the ROS-scavenging action of Ex-4. Furthermore, H_2_O_2_ alone consumed large amounts of antioxidants and produced more MDA, a reliable marker of oxidative damage. However, Ex-4 pretreatment restored the homeostasis of SOD, GSH and GPX but suppressed MDA production (Fig. 6B–E). SOD, GSH and GPX are the most important antioxidant factors; they reduce oxidative injury by counteracting excessive oxidants, restoring normal redox levels in cells. This information illustrates that the ROS-reducing effect of Ex-4 was due to the enhancement of intracellular antioxidants. However, LY294002 treatment inhibited the ROS-scavenging action of Ex-4, indicating that Ex-4 played a vital role in improving the antioxidant defense system that removes excessive H_2_O_2_ and reduces the risk of oxidative injury via the PI3K/Akt pathway in MSC.

It has been shown that, when attacked by devastating ROS, mitochondria collapse and activate caspase9/3, initiating and amplifying apoptotic signals; this sequence of events is termed the mitochondrial death pathway^28^,^29^. The loss of ΔΨm is believed to be one of the initiating factors of the mitochondrial apoptosis pathway^30^. In our study, we found that H_2_O_2_ led to lower ΔΨm, as evidenced by immunofluorescence and flow cytometry (Fig. 6F). Normal cells stained with a ΔΨm-sensitive dye (JC-1) exhibited red fluorescence, which is indicative of coupled mitochondria with a normal ΔΨm. However, when the ΔΨm is low, JC-1 becomes a monomer with green fluorescence. Furthermore, H_2_O_2_-MSC with lower ΔΨm expressed more caspase9 and cleaved caspase3 (Fig. 6G), suggesting that H_2_O_2_ may induce MSC apoptosis via the mitochondrial death pathway. By contrast, the Ex-4-induced MSC had a higher ΔΨm and less caspase9/3. Additionally, Akt inhibition suppressed the protective effects of Ex-4, suggesting that Ex-4 had a role in preserving ΔΨm and inhibiting mitochondria-mediated apoptosis through Akt activation. Furthermore, we found that Ex-4 treatment increased the anti-apoptotic proteins Bcl-2, c-IAP1/2, and survivin, whereas it reduced the pro-apoptotic proteins Bax and Bad (Fig. 6G). The balance between Bad/Bax and Bcl-2 regulates mitochondrial membrane integrity and ∆Ψm stabilization; more importantly, c-IAP and survivin are inhibitors of caspase9 and, subsequently, apoptosis^31^,^32^. These data indicated that Ex-4 could maintain the ΔΨm by increasing anti-apoptotic proteins, which halt the progression of mitochondrial apoptosis. However, the protective action of Ex-4 vanished in the presence of an Akt inhibitor, suggesting that the PI3K/Akt pathway may be the major anti-apoptotic downstream signal of Ex-4.

## Discussion

Evidence^33^,^34^,^35^ has accumulated for the role of MSC grafts in the treatment of myocardial infarction due to their myocardial replacement, cardioprotective, and cardiotrophic effects^36^. Despite the observed beneficial effects of cell therapy, the retention^37^, survival^7^ and functionality of engrafted cells^38^ still need to be improved. It has been reported that 99% of transplanted cells are lost after transplantation^39^,^40^ because of cellular apoptosis, nutrient deprivation and the inflammatory response^41^. Furthermore, the absence of cell trafficking and/or homing factors in transplanted cells contributes to the “washout” of engrafted cells from the heart and their migration to distant organs^9^. In parallel, we have observed that the numbers of MSC in bone marrow are very low, and their self-renewal capacity is limited, especially in the severe resident microenvironment^5^,^42^, which presents a critical issue — how to harvest a sufficient number of cells for transplantation. Therefore, we investigated the effects of Ex-4 on MSC proliferation, migration and H_2_O_2_-induced apoptosis.

Ex-4, an antidiabetic agent originally isolated from the venom of the Gila monster lizard, shares 53% amino acid sequence identity with human GLP-1^43^. Therefore, Ex-4 exhibits biologic actions similar to those of GLP-1, and these effects are exclusively mediated through GLP-1R-dependent and -independent pathways^19^,^20^. As Ex-4 acts on cells, three main intracellular signaling pathways (cAMP/PKA, PI3K/Akt, and MAPK) are activated^44^. In our study, we confirmed that Ex-4 was an upstream activator of PI3K/Akt. In addition, Ex-4 increased MSC proliferation in a time- and dose-dependent manner. The number of cells in the S phase of the cell cycle and the lifespan of MSC significantly increased under Ex-4 exposure. The molecular mechanisms may be associated with the activation of the Cyclin D1/E—p-Rb pathway, which contributed to the release of the transcription factor E2F-1 in MSC, leading to the expression of genes required for DNA synthesis. However, Akt inhibition reversed these effects of Ex-4 on cell cycle protein expression and MSC growth. Ex-4 also increased CXCR4 expression and the subsequent migration response via the PI3K/Akt pathway^45^,^46^. Although CXCR4 is highly expressed by MSC within the bone marrow, its expression is markedly reduced during the *ex vivo* expansion of MSC^47^,^48^, which reduces their ability to respond to homing signals emanating from injured sites. In our study, under normal conditions, the number of CXCR4^+^ cells was low to undetectable in MSC at passage 3. However, Ex-4 increased the percentage of CXCR4^+^ cells, which was responsible for the enhanced migration response evidenced by the transwell and wound-healing assays. Thus, we have provided a simple and feasible means to improve the numbers of CXCR4^+^ cells during *ex vivo* expansion. These results illustrate that Ex-4 could be considered an adjuvant to improve the biological functions of MSC, especially their proliferation and migration. This procedure offers a new way to acquire plentiful numbers of engrafted MSC containing a higher proportion of the CXCR4^+^ subgroup. However, we must admit that the percentage of CXCR4^+^ cells after Ex-4 treatment (20nM) is not very high (18.46 ± 1.33%), although there was an obvious trend toward an increase after Ex-4 incubation. In light of the important role of SDF-1/CXCR4 on MSC homing to infarcted myocardium, other methods should be introduced along with Ex-4 to further improve the proportion of CXCR4^+^ cells.

Although the increased proliferative capacity and migration response of MSC may contribute to higher transplantation efficiency in clinical applications, the hostile environment of injured heart tissue, including hypoxia and oxidative stress, causes excessive cell death^49^, leading to an urgent need to enhance the resistance of MSC to apoptosis. Therefore, we explored the pro-survival effect of Ex-4 on MSC under oxidative stress induced by H_2_O_2_. The results showed that H_2_O_2_ induced higher intracellular ROS, lower mitochondrial ΔΨm and more cellular apoptosis. However, Ex-4 pretreatment could indirectly reduce the excessive ROS and preserve mitochondrial function, which contributed to the inhibition of mitochondria-mediated apoptosis under H_2_O_2_. It has been demonstrated that cells can normally defend themselves against ROS damage through the use of specific ROS-reducing mechanisms, which may be enzymatic (involving dismutases, catalases, and peroxidases) or non-enzymatic (involving vitamins A, C and E, urate, and bilirubin). In our study, Ex-4 was capable of restoring SOD, GSH, and GPX levels as well as decreasing MDA production. SOD, GSH and GPX are important intracellular antioxidant mediators that interact with superfluous ROS and balance the status of oxidation. MDA is a reliable marker of the degree of oxidative injury, and the lower MDA after Ex-4 pretreatment indicated the near-normal redox levels in MSC under H_2_O_2_. This information suggested that Ex-4 played a role in regulating the intrinsic antioxidant repair system to indirectly reduce intracellular ROS and prevent any accumulation of cellular damage. Moreover, Ex-4 could reverse the loss of mitochondrial ΔΨm induced by H_2_O_2_ through the upregulation of c-IAP/Bcl-2/survivin and the downregulation of Bax/Bad. The lower ΔΨm under H_2_O_2_ indicated the dysfunction of the electron transport chain in mitochondria, leading to more ROS production, which in turn aggravated oxidative stress^50^ and/or activated the caspase9-mediated mitochondrial death pathway^29^. Ex-4 treatment increased Bcl-2 expression but reduced Bax expression, which maintained mitochondrial membrane integrity and ΔΨm stabilization. Additionally, the higher c-IAP/Bcl-2/survivin levels under Ex-4 may suppress mitochondrial death pathways by inactivating cytochrome c and caspase9, which are stimulators of caspase3^51^,^52^,^53^. Taken together, these results indicate that Ex-4 could balance the expression of anti- and pro-apoptotic proteins to preserve ΔΨm and subsequently inhibit the mitochondrial apoptosis pathway. Additionally, the indirect ROS-scavenging effect of Ex-4 is involved in ΔΨm stabilization and cellular survival because excessive levels of ROS have toxic effects on mitochondrial function and structure^54^. The reduced ROS content associated with Ex-4 would result in a drop in the severity of oxidative stress, especially reducing the levels of mitochondrial damage, which improves MSC resistance and minimizes the risk of cellular apoptosis. In conclusion, we confirmed that Ex-4 could improve MSC vitality under oxidative stress by inhibiting mitochondrial death pathways and enhancing antioxidant defense systems to directly remove ROS. Notably, we also provided a therapeutic target, mitochondria, which are vital to the initiation and transformation of cellular apoptosis signals; the protection of mitochondrial function and the inactivation of the caspase9-related mitochondrial death pathway to improve cellular survival after transplantation may be the focus of MSC-based therapies in the future.

The anti-apoptotic effects of Ex-4 on ADSCs were reported to be mediated via the PI3K/Akt-STAT3 pathway by our team^26^,^55^. Moreover, PI3K/Akt also plays an important role in the biological behavior of MSC^22^. In the present study, Ex-4 was revealed to be an upstream activator of Akt, and Akt inhibition abolished the promoting effects of Ex-4 on the growth, CXCR4 expression and mobilization of MSC. Furthermore, a blockade of the Akt pathway caused a reduction in anti-apoptotic proteins and an increase in cellular apoptosis. Together, these data revealed that the Akt pathway is necessary for the protective role of Ex-4 in MSC proliferation, migration and survival. We also found that inhibiting Akt by LY294002 could not completely suppress the promoting effects of Ex-4 on MSC, suggesting that other pathways may be involved in the actions of Ex-4. Several studies have argued that cAMP/PKA, PI3K/Akt and MAPK could be activated by Ex-4; whether these pathways also contribute to the beneficial influence of Ex-4 requires further research. Recent studies have found that Akt activation in MSC induced the accumulation of Secreted frizzled-related protein 2 (Sfrp2)^56^, which increased MSC self-renewal via the inhibition of both the Wnt and BMP pathways^57^ and protected MSC from apoptosis by promoting cytoplasmic β-Catenin translocation into the nucleus^58^. These are possible mechanisms of the PI3K/Akt pathway effects on MSC proliferation and apoptosis. Moreover, Akt activation has also been demonstrated to cause a marked increase in CXCR4 expression^59^ and migratory behavior^60^ under low-oxygen conditions; the underlying mechanism may be associated with the activation of hypoxia-inducible factor-1, which activates the expression of relevant genes. However, whether Sfrp2 is involved in the enhanced CXCR4 expression and migration requires further study. Together, we can conclude from these data that the PI3K/Akt pathway is a vital signal that improves MSC transplantation potency by overcoming the conflict between limited proliferation, poor migration and excessive apoptosis before and after engraftment. Therefore, approaches to activate the PI3K/Akt pathway could be the focal point to increase the efficiency of MSC-based transplantation therapies in the future. In this study, we identified an easy and practical means of enhancing Akt expression and MSC biological function: Ex-4. Importantly, given that Ex-4 itself exerts cardioprotective activity, as evidenced by numerous animal tests and clinical trials^61^,^62^,^63^,^64^, the combination of Ex-4 and MSC for the treatment of AMI may be a promising therapeutic modality in the future. Nonetheless, additional insights into such a combination should first be obtained to provide solid evidence for clinical applications.

## Additional Information

**How to cite this article**: Zhou, H. *et al.* Effects of Exendin-4 on bone marrow mesenchymal stem cell proliferation, migration and apoptosis *in vitro*. *Sci. Rep.* **5**, 12898; doi: 10.1038/srep12898 (2015).
